# Supplementary material for: Identification of ABHD6 as a lysophosphatidylserine lipase in the mammalian liver and kidneys
Source: J Biol Chem. 2025 Jan 4;301(2):108157. doi: 10.1016/j.jbc.2025.108157 (PMC11808726; doi:10.1016/j.jbc.2025.108157)
Supplement: Supplementary Figure [file mmc1.pdf]

SUPPLEMENTARY FIGURES

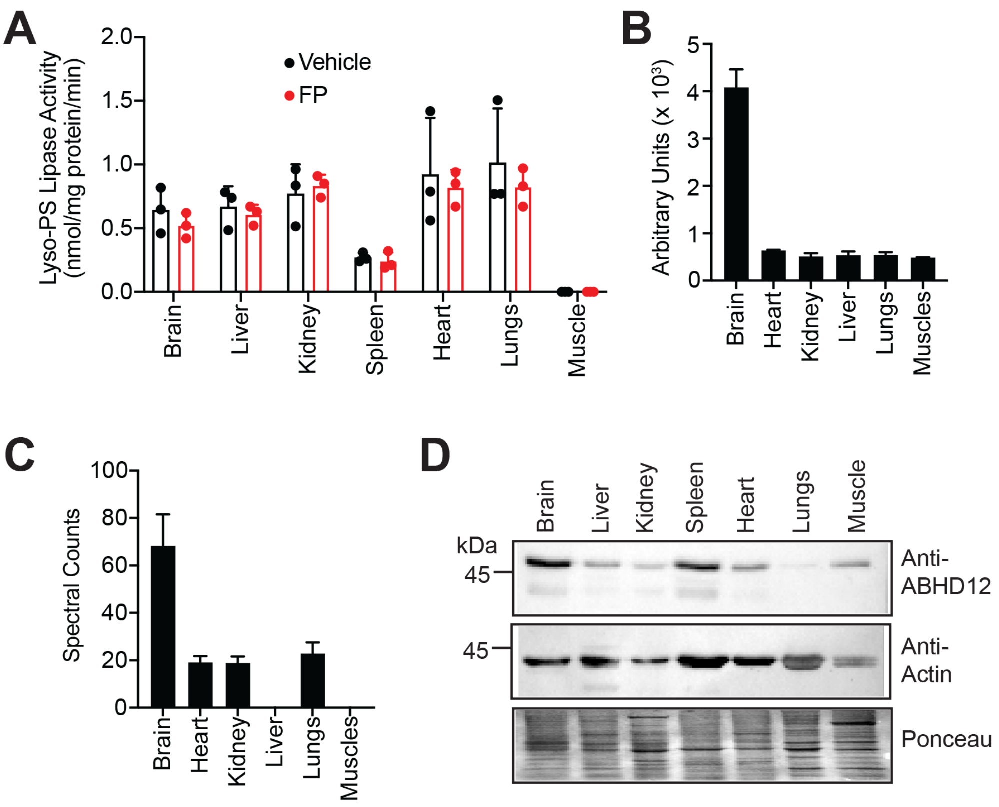

Supplementary Figure 1. ABHD12 controls lyso-PS levels only in the mouse brain. (**A**) The lyso-PS lipase activity in the soluble fractions of different mouse tissues treated with vehicle (DMSO) or FP-rhodamine (20 μM, 45 min). All assays were done using 20 μg of proteome against 100 μM C17:1 lyso-PS for 30 min at 37 °C. (**B**) The mRNA expression data exported from the large-scale gene expression database BioGPS (1,2), showing the heightened expression of ABHD12 in the mouse brain. (**C**) The activity of ABHD12 in various mouse tissues from a publicly available LC-MS based ABPP dataset (spectral counting-based data) (3), showing the heightened activity of ABHD12 in the membrane fraction of the mouse brain. (**D**) A representative western blot, showing the tissue distribution of ABHD12, further confirming highest protein levels on ABHD12 in the membrane fraction of the mouse brain consistent with data from (**B**) and (**C**). 50 μg of each membrane fraction was loaded onto the gel, and both β-Actin and Ponceau S staining were used as loading controls for this experiment. This immunoblotting study was done three times with reproducible result each time. (All bar plots presented in this figure represents mean ± standard deviation from two (for **B**) or three (**A** and **C**) biological replicates.

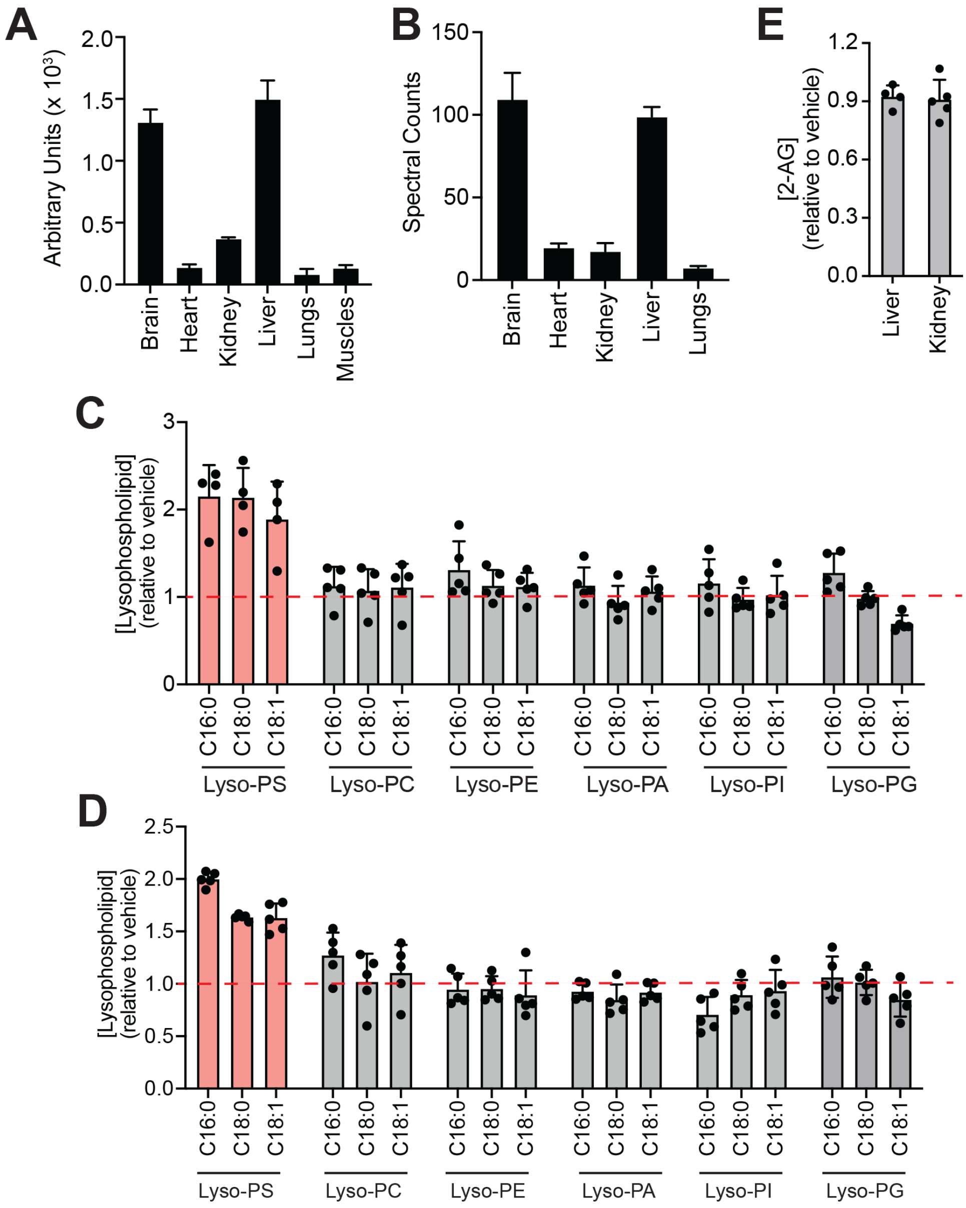

Supplementary Figure 2. ABHD6 is a major lyso-PS lipase in the liver and kidney. (**A**) The mRNA expression data exported from the large-scale gene expression database BioGPS (1,2), showing the heightened expression of ABHD6 in the mouse brain and liver, and to a lesser extent in the kidney. (**B**) The activity of ABHD6 in various mouse tissues from a publicly available LC-MS based ABPP dataset (spectral counting-based data) (3), showing the heightened activity of ABHD12 in the membrane fraction of the mouse brain and liver. (**C, D, E**) Relative levels of different lysophospholipids in the liver (**C**), and kidney (**D**) and 2-AG (**E**), upon KT185 treatment (10 mg/kg body weight, 4 hours), showing an increase of only lyso-PS, but no other lysophospholipid, from the selective inhibition of ABHD6. All bar plots presented in this figure represents mean ± standard deviation from two (for **A**) or four or more (**C, D** and **E**) biological replicates

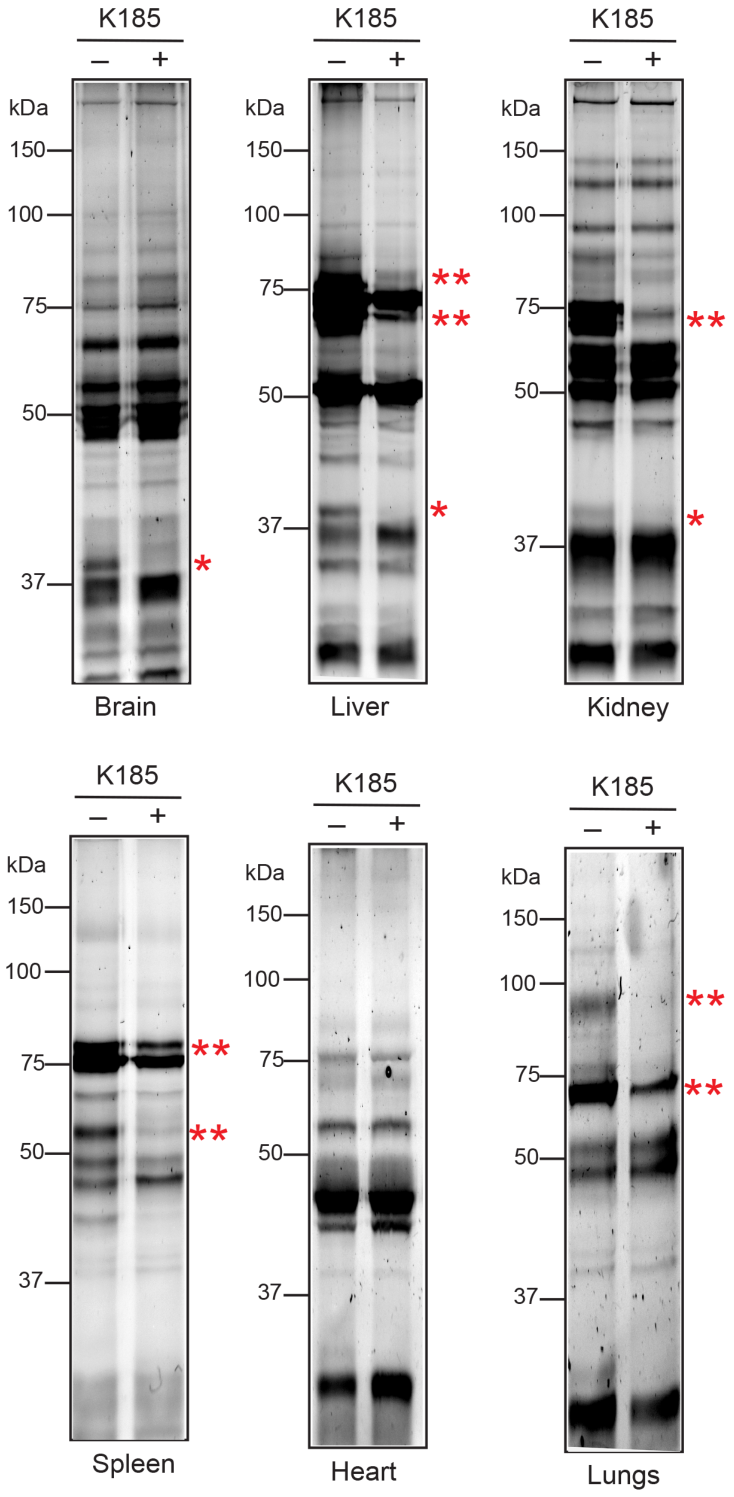

Supplementary Figure 3. Complete ABPP gels from **Figure 6A**, showing the loss of ABHD6 activity (band corresponding to the single red asterisk) in the membrane fractions of the brain, liver and kidney upon treatment with KT185 (orally, 10 mg/kg body weight, 4 hours). No ABHD6 activity (at ~ 37 kDa) was detected in the membrane fractions of the spleen, heart and lungs. The off-targets of KT185 (bands corresponding to two red asterisk) seen in the liver, kidney, spleen and lungs are likely enzymes from the CES-family (CES2 and CES3).

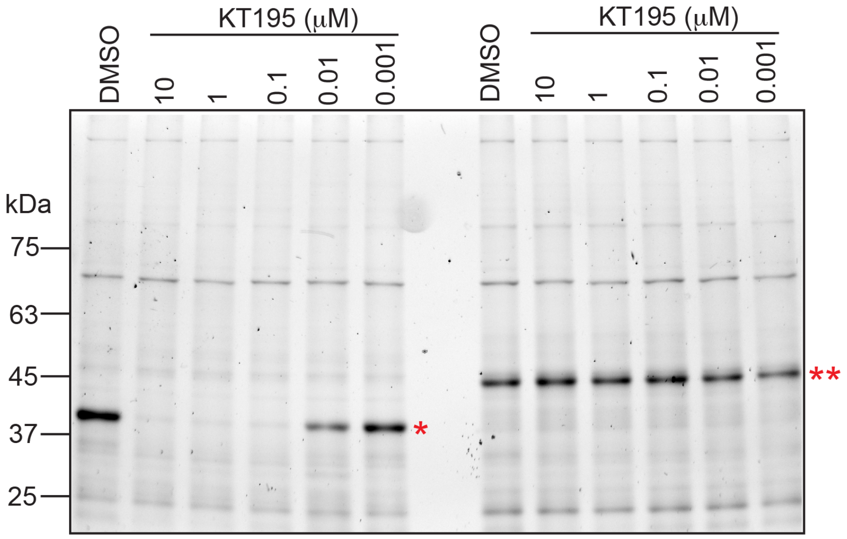

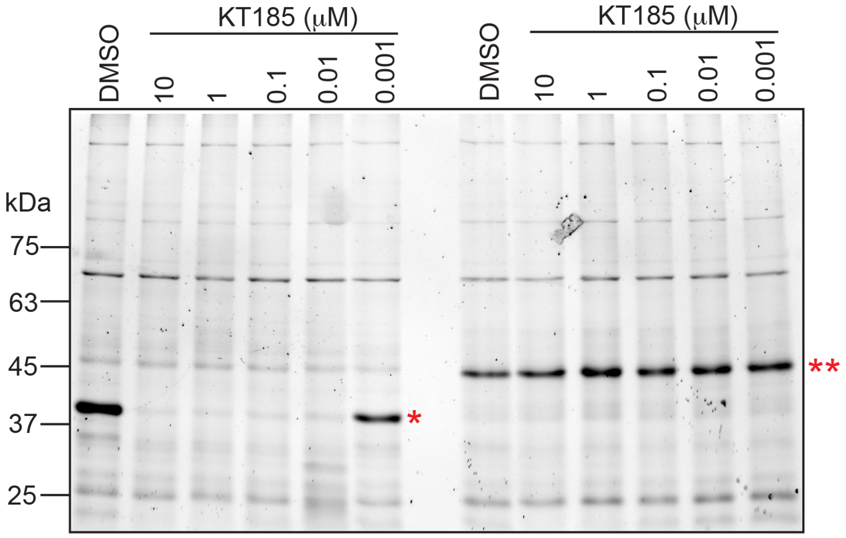

Supplementary Figure 4. KT195 and KT185 are ABHD6 selective inhibitors. Consistent with published literature (4,5), we find that both KT195 and KT185 selectively and potently inhibit WT ABHD6 (IC_{50} < 10 nM) (single red asterisk), but not WT ABHD12 (IC_{50} > 10 μM) (double red asterisk) by gel-based ABPP analysis. All gel-based ABPP assays were done in membrane fractions of HEK293T cells overexpressing WT ABHD6 or WT ABHD12. This gel-based ABPP experiment was repeated three times with reproducible results each time.

REFERENCES.

1. Wu, C., Jin, X., Tsueng, G., Afrasiabi, C., and Su, A. I. (2016) BioGPS: building your own mash-up of gene annotations and expression profiles. *Nucleic Acids Res* **44**, D313-316

2. Wu, C., Orozco, C., Boyer, J., Leglise, M., Goodale, J., Batalov, S., Hodge, C. L., Haase, J., Janes, J., Huss, J. W., 3rd, and Su, A. I. (2009) BioGPS: an extensible and customizable portal for querying and organizing gene annotation resources. *Genome Biol* **10**, R130

3. Bachovchin, D. A., Ji, T., Li, W., Simon, G. M., Blankman, J. L., Adibekian, A., Hoover, H., Niessen, S., and Cravatt, B. F. (2010) Superfamily-wide portrait of serine hydrolase inhibition achieved by library-versus-library screening. *Proc Natl Acad Sci U S A* **107**, 20941-20946

4. Hsu, K. L., Tsuboi, K., Chang, J. W., Whitby, L. R., Speers, A. E., Pugh, H., and Cravatt, B. F. (2013) Discovery and optimization of piperidyl-1,2,3-triazole ureas as potent, selective, and in vivo-active inhibitors of alpha/beta-hydrolase domain containing 6 (ABHD6). *J Med Chem* **56**, 8270-8279

5. Hsu, K. L., Tsuboi, K., Adibekian, A., Pugh, H., Masuda, K., and Cravatt, B. F. (2012) DAGLbeta inhibition perturbs a lipid network involved in macrophage inflammatory responses. *Nat Chem Biol.* **8**, 999-1007
